# Improving the design stage of air pollution studies based on wind patterns

**DOI:** 10.1038/s41598-022-11939-6

**Published:** 2022-05-13

**Authors:** Léo Zabrocki, Anna Alari, Tarik Benmarhnia

**Affiliations:** 1 Paris School of Economics and École des Hautes Etudes en Sciences Sociales, 48 Boulevard Jourdan, 75014 Paris, France; 2 Barcelona Institute for Global Health (ISGlobal), Carrer del Rosselló, 132, 08036 Barcelona, Spain; 3 Department of Family Medicine and Public Health, Scripps Institution of Oceanography, University of California, 8622 Kennel Way, La Jolla, San Diego, CA USA

**Keywords:** Environmental impact, Epidemiology, Environmental economics

## Abstract

A growing literature in economics and epidemiology has exploited changes in wind patterns as a source of exogenous variation to better measure the acute health effects of air pollution. Since the distribution of wind components is not randomly distributed over time and related to other weather parameters, multivariate regression models are used to adjust for these confounding factors. However, this type of analysis relies on its ability to correctly adjust for all confounding factors and extrapolate to units without empirical counterfactuals. As an alternative to current practices and to gauge the extent of these issues, we propose to implement a causal inference pipeline to embed this type of observational study within an hypothetical randomized experiment. We illustrate this approach using daily data from Paris, France, over the 2008–2018 period. Using the Neyman–Rubin potential outcomes framework, we first define the treatment of interest as the effect of North-East winds on particulate matter concentrations compared to the effects of other wind directions. We then implement a matching algorithm to approximate a pairwise randomized experiment. It adjusts nonparametrically for observed confounders while avoiding model extrapolation by discarding treated days without similar control days. We find that the effective sample size for which treated and control units are comparable is surprisingly small. It is however reassuring that results on the matched sample are consistent with a standard regression analysis of the initial data. We finally carry out a quantitative bias analysis to check whether our results could be altered by an unmeasured confounder: estimated effects seem robust to a relatively large hidden bias. Our causal inference pipeline is a principled approach to improve the design of air pollution studies based on wind patterns.

## Introduction

A growing literature in economics and epidemiology has recently re-examined the short-term effects of air pollution on mortality and emergency admissions using causal inference methods. Among these techniques, instrumental variable strategies have been very popular since they can overcome the biases caused by unmeasured confounders and measurement errors in air pollution exposure^1–6^. Daily changes in wind directions are such instrumental variables since they arguably meet two of the three main requirements for the method to be valid: they can strongly affect air pollutant concentrations while having no direct effects on health outcomes^7–9^. This strategy however rests on the remaining assumption that changes in wind directions occur randomly, which is often not credible without further statistical adjustments. One could unfortunately fear that the resulting analysis would depend on the quality of the model^10,11^. Does the model take into account all relevant confounding factors, and if so, are they adjusted for with the correct functional forms? Is the model also able to extrapolate when there is little overlap in covariate distributions?

To illustrate these issues, imagine that we are interested in estimating the influence of particulate matters on daily mortality in Paris, France, over the 2008–2018 period. Research in atmospheric science has shown that winds blowing from the North-East could transport particulate matters due to wood burning in the region but also from other sources located in North-Eastern Europe^12–14^. We could therefore use the comparison of winds blowing from the North-East to those from other directions as an instrumental variable for particulate matters.

**Figure 1:**
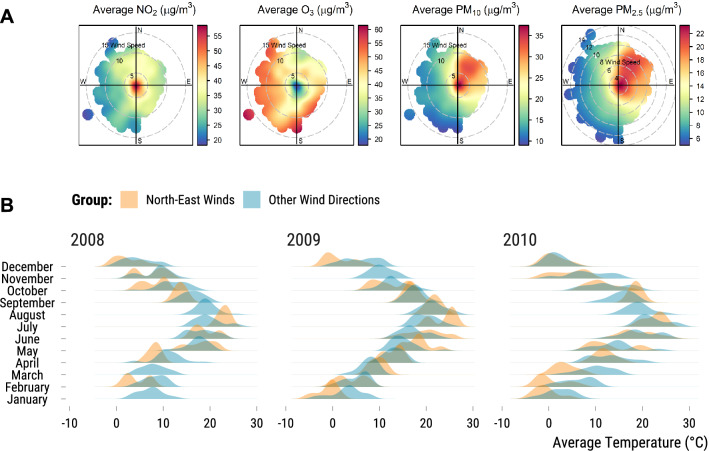
Polar plots of air pollutant concentrations predicted by wind components and average temperature imbalance of wind directions by year and month. In panel (**A**), each plot represents the concentrations (in ${\upmu \hbox {g}/\hbox {m}^3}$) of an air pollutant that were predicted using a generalized additive model based on a smooth isotropic function of the two wind components *u* and *v*^15^. The direction from which the wind blows is described on a 360$^\circ$ compass rose and wind speed (in m/s) is represented by a series of increasing circles starting from the intersection of the two cardinal directions axes where wind speed is null: the farther the circle is away from the intersection, the faster the wind speed is. In panel (**B**), the density distribution of the average temperature (in $^\circ$C) is drawn for North-East winds (orange colour) and other wind directions (blue colour). The figure is divided into subplots by month and year (2008–2010).

In Panel A of Fig. 1, we display polar plots of air pollutant concentrations that were predicted using a Generalized Additive Model (GAM) and wind components as inputs^15^. We clearly see that winds blowing from the North-East are associated with higher PM$_{10}$ and PM$_{2.5}$ concentrations. These patterns could however be confounded by other variables such as the weather parameters or a shared seasonality in air pollution and wind patterns. For instance, in Panel B of Fig. 1, the density distribution of the average temperature ($^\circ$C) is not similar for the groups of wind directions. We must take into account this confounding variable if we want to make the as-if random distribution of North-East wind more credible. Multivariate linear regression have been the standard approach to help achieve this goal but more flexible methods such as generalized additive models and machine learning algorithms could also be used^16,17^. Yet, even a very flexible model will not overcome the second issue visible in Panel B of Fig. 1: as for January 2008, the model will sometimes depend on extrapolation since there are no empirical counterfactuals to estimate what would have happened had the wind blown from the North-East. Finally, it could be argued that we fail to adjust for a confounding variable which we have not measured. In addition to explaining with qualitative arguments why it is not likely the case, we should also try to quantify the bias induced by an unmeasured confounder.

In this paper, we show how we can evaluate the extent to which studies exploiting wind directions as instrumental variables could be prone to the issues raised above. To achieve this goal, we follow the four consecutive stages of the causal inference pipeline proposed by^18,19^ that explicitly embed the design of this type of observational study within an hypothetical randomized experiment^20–23^.

First, in a *conceptual stage*, we clearly state the causal question of interest using the Neyman–Rubin potential outcomes framework^24,25^. Our treatment of interest is the effect of North-East winds on air pollution compared to other wind directions. To estimate this effect, for treated days with winds blowing from the North-East, we need to impute the concentrations that would have been observed had winds blown from other directions. The issue is that wind patterns are not randomly assigned: control days with wind blowing from other directions are not similar to treated days.

We therefore implement a *design stage* where we approximate a pairwise randomized experiment using a matching algorithm recently designed for air pollution studies^26^. Matching is a transparent method to adjust for confounders without making parametric assumption and directly looking at observed outcomes^27,28^. Given a set of chosen covariate distances, each treated day is matched to its closet control day. This method also avoids model extrapolation since treated days for which no control days exist in the data are discarded from the analysis.

The third step is an *analysis stage* where we estimate the influence of North-East winds on air pollutant concentrations. We simply compute the average difference in concentrations between matched treated and control days and rely on Neymanian inference to compute an estimate of the sampling variability^22^. The last and fourth step is to carry out a *sensitivity analysis*. Throughout the previous steps, we must make the strong assumption that no unmeasured variables could be related both to wind patterns and air pollutant concentrations. Quantitative bias analysis was initially proposed by^29^ to assess which magnitude of hidden bias would be required to alter observed results. We follow here the method developed by^21,30^.

With this study, we aim to bring two contributions to the causal inference literature on the acute health effects of air pollution. First, we show that using wind directions as instrumental variables requires more caution to make the assumption that they are “as-if” randomly distributed according to observed covariates convincing. The effective sample size where treated and control units are similar on a set of observed covariates is actually small. The standard approach used in the literature based on multivariate regression models will therefore rely on its ability to adjust correctly for the functional forms of covariates and extrapolate to units without empirical counterfactuals. Second, our quantitative bias analysis reveals that the estimated increase in particulate matter concentrations due to North-East winds is relatively robust to the presence of hidden bias. Even if an unobserved confounding factor is twice more common among days with winds blowing from the North-East than among days with winds from other directions, the large range of estimates consistent with the data remains positive.

We also hope that the approach we propose in this paper could be of interest to atmospheric scientists. The fact that wind patterns play a key role in the variation of air pollution concentrations is obviously not new^31–34^. Yet, causal inference methods have rarely been implemented in atmospheric science to estimate the influence of weather parameters on air pollution. We believe that mimicking a randomized experiment corresponds to an intuitive approach and could complement source apportionment and emission inventory approaches. While wind is non manipulable, emission sources are and our framework could also serve as a stepping-stone to evaluate potential interventions to control emissions—if a source is shut-down in the North-East of Paris, would wind blowing from this direction influence less specific air pollutant concentrations?

We took great care to make our work fully reproducible to help researchers implement but also improve and criticize our approach. Data and detailed **R** codes are available at https://lzabrocki.github.io/design_stage_wind_air_pollution/ and backed-up in an Open Science Framework repository^35^.

## Methods

### Data

We built a dataset combining daily time series of air pollutant concentrations and weather parameters in Paris over the 2008–2018 period. We chose to carry out an analysis at the daily level as done in studies on the acute health effects of air pollution^3,4,6^.

**Figure 2:**
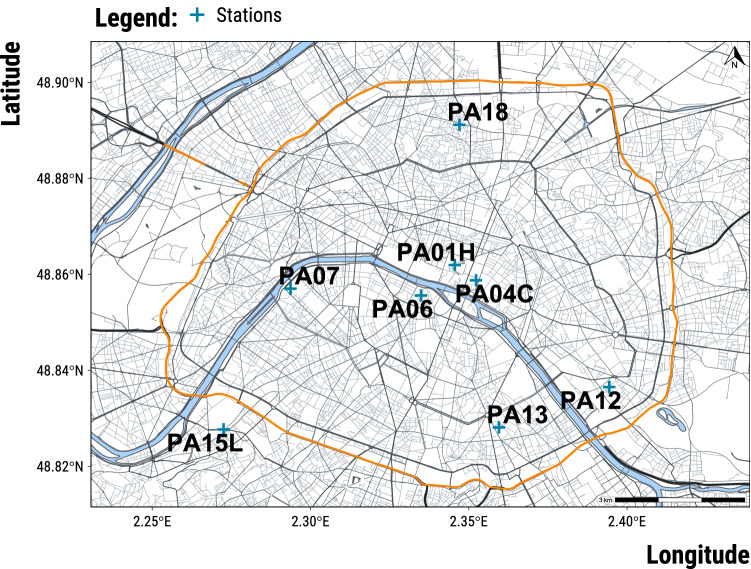
Map of road network and location of air pollution measuring stations in Paris, France . Grey lines represent the road network. The orange line is the orbital ring surrounding Paris. Blue crosses are the locations of air pollution measuring stations. NO$_{2}$ concentrations are measured at stations PA07, PA12, PA13, PA18; O$_{3}$ concentrations at PA13, PA18; PM$_{10}$ at PA18; PM$_{2.5}$ at PA01H and PA04C. The map was created with the R programming language (version 4.1.0)^36^, data were provided by OpenStreetMap^37^ and retrieved with the osmdata package^38^.

First, we obtained hourly air quality data from AirParif, the local air quality monitoring agency. Figure 2 displays the location of the selected measuring stations. Using a 2.5% trimmed mean, we first averaged at the daily level the concentrations (${\upmu \hbox {g}/\hbox {m}^3}$) of background measuring stations for NO$_{2}$, O$_{3}$ and PM$_{10}$. For a given day, if more than 3 hourly readings were missing, the average daily concentration was set to missing. The proportion of missing values for stations ranged from 2.8% up to 9.1%. We also computed the average daily concentrations of PM$_{2.5}$ but 25% of the recordings were missing: the air pollutant was not measured by Airparif between 2009/09/22 and 2010/06/23. It is important to note that we did not retrieve data from traffic monitors but only from background monitors as they are used to assess the residential exposure of a city population in epidemiological studies.

We then retrieved meteorological data from the single monitoring station located in the South of the city and ran by the French national meteorological service Météo-France. We extracted daily observations on wind speed (m/s), wind direction (measured on a 360$^\circ$ wind rose where 0$^\circ$ is the true North), the average temperature ($^\circ$C), and the rainfall duration (min). Weather parameters had very few missing values (e.g., at most 2.5% of observations were missing for the rainfall duration).

Finally, to avoid working with a reduced sample size, we imputed missing values for all variables but PM$_{2.5}$. There were no clear patterns in the missingness of NO$_{2}$, O$_{3}$ and PM$_{10}$ concentrations. We used the chained random forest algorithm implemented by the **R** package missRanger^39^. A small simulation exercise showed that it had good performance for imputing NO$_{2}$ concentrations (the absolute difference between observed and imputed values was equal to 3.2 $\upmu \hbox {g}/\hbox {m}^{3}$ for an average concentration of 37.6 $\upmu \hbox {g}/\hbox {m}^{3}$) but was much less effective for imputing PM$_{10}$ concentrations (the absolute difference between observed and imputed values was equal to 6.1 $\upmu \hbox {g}/\hbox {m}^{3}$ for an average concentration of 23.4 $\upmu \hbox {g}/\hbox {m}^{3}$). Once the data were imputed, we averaged the air pollutant concentrations at the city level as it is the spatial level of analysis used in^3,4^.

Further details on data wrangling and an exploratory analysis of the data can be found in the supplementary materials (https://lzabrocki.github.io/design_stage_wind_air_pollution, tab Data). We were not allowed to share weather data from Météo-France so we added some noise to the weather parameters.

### A causal inference pipeline

We present below the four stages of the causal inference pipeline we advocate to use for improving the design of air pollution studies based on wind patterns. Its implementation was done with the R programming language (version 4.1.0)^36^.

#### Stage 1: Defining the treatment of interest

The first step of our causal inference approach is to clearly state the question we are trying to answer: *What is the effect of North-East winds on particulate matter in Paris over the 2008–2018 period?* This question is motivated by the exploratory analysis of Fig. 1 and research in atmospheric science on the sources of particulate matter located in the North-East of the city. Our treatment of interest is therefore defined as the comparison of air pollutant concentrations when winds are blowing from the North-East (10$^\circ$–90$^\circ$) with concentrations when wind come from other directions. We frame this question in the Rubin–Neyman causal framework^24,25^. Our units are 4018 days indexed by *i* (*i*=1,..., I). For each day, we define our treatment indicator W$_i$ which takes two values. It is equal to 1 if the unit is treated (the wind blows from the North-East), and 0 if the unit belongs to the control group (the wind is blowing from another direction). Under the Stable Unit Treatment Value Assumption (STUVA), we assume that each day can have two potential concentrations in $\upmu \hbox {g}/\hbox {m}^{3}$ for an air pollutant: Y$_{i}$(1) if the wind blows from the North-East and Y$_{i}$(0) if the wind blows from another direction.

The fundamental problem of causal inference states that we can only observe for each day one of these two potential outcomes: it is a missing data problem^40,41^. The observed concentration of an air pollutant Y$^{\text {obs}}$ is defined as Y$^{\text {obs}}$ = (1-W$_{i}$) $\times$ Y$_{i}$(0) + W$_{i}$ $\times$ Y$_{i}$(1). If the unit is treated, we observe Y$_{i}$(1). If it is a control, we observe Y$_{i}$(0). To estimate the effect of North-East winds on air pollutant concentrations, we therefore need to impute the missing potential outcomes of treated units—what would have been the air pollutant concentrations if the wind had blown from another direction?

#### Stage 2: Designing the hypothetical randomized experiment

**Figure 3:**
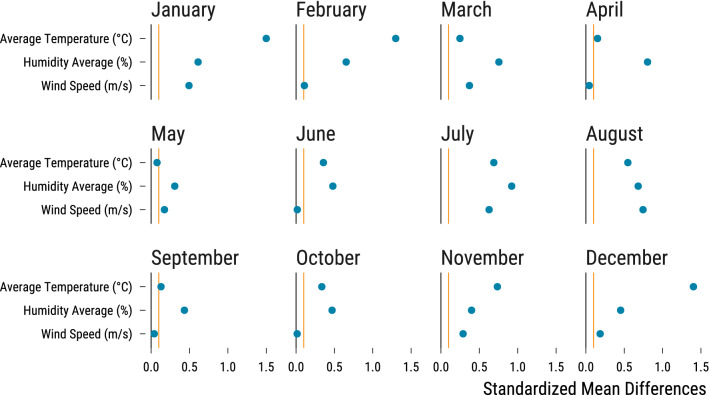
Evidence of imbalance for weather covariates . For each month, we compute the absolute standardized differences for continuous weather covariates between treated and control groups. These differences are represented as blue points. The vertical orange line is the 0.1 threshold which is used in the matching literature to spot covariates imbalance. The vertical black line is at 0.

The second stage of our causal inference pipeline is to embed our non-randomized study within an hypothetical randomized experiment. We are dealing with an observational study where North-East winds are not randomly distributed through a year and are correlated with other weather parameters influencing air pollutant concentrations. In Fig. 3, we plot, for each month, the absolute standardized mean differences between treated and control units for the average temperature, relative humidity and wind speed: most differences are superior to 0.1, which is often considered as a threshold to assess the imbalance of covariates.

To better approximate a randomized experiment, we must therefore find the subset of treated units which are similar to control units. Formally, we want to make plausible for this subset of units the assumption that the treatment assignment is independent from the potential outcomes of units given their covariates **X**: Pr(**W** | **X**, **Y**(0), **Y**(1)) = Pr(**W** | **X**). The issue is that some units’ covariates are observed while other are not. Unlike a randomized experiment where both observed and unobserved covariates will be, on average, balanced across treatment and control groups, we must assume that no unobserved covariates affect the treatment assignment.

Matching methods are particularly convenient to design hypothetical randomized experiments. Contrary to standard regression approaches, matching is a non-parametric way to adjust for observed covariates while avoiding model extrapolation since units without counterfactuals in the data are discarded from the analysis. Specifically, we use a constrained matching algorithm to design a pairwise randomized experiment where, for each pair, the probability of receiving the treatment is equal to 0.5 (see^26^ for further details on the algorithm). Each treated unit is matched to its closest unit given a set of covariate constraints which represent the maximum distance, for each covariate, allowed between treated and control units. We match on the two sets of covariates influencing both wind directions and air pollutant concentrations.

First, we match on calendar variables such as the Julian date, weekend, holidays and bank days indicators. A treated unit could be matched up to a control unit with a maximum distance of 60 days. If we extend this distance, it would be easier to match treated units to control units but the treatment effect could be biased by seasonal variation in air pollutant concentrations. We match exactly treated and control units for the other calendar indicators.

Second, we match on weather variables. The average temperature between treated and control units could not differ by more than 5$^\circ$. The difference in wind speed must be less than 0.5 m/s. The rainfall duration (divided in four ordinal categories) needs to be the same and the absolute difference in average humidity could be up to 12 percentage points. We also force the absolute difference in PM$_{10}$ concentrations in the previous day to be less or equal to 8 $\upmu \hbox {g}/\hbox {m}^{3}$. The thresholds we set up were chosen through an iterative process were we checked (1) that they led to balanced sample of treated and control units and (2) that there were enough matched pairs to draw our inference upon.

Finally, the Stable Unit Treatment Value Assumption (SUTVA) requires that there is no interference between units and no hidden variation of the treatment. To make this assumption more plausible, we discard from the analysis the matched pairs for which the distance in days is inferior to 4 days and make sure that the first lag of the treatment indicator for treated and control units.

#### Stage 3: Analyzing the experiment using Neymanian inference

In the third stage, we proceed to the analysis of our hypothetical pairwise randomized experiment. Several modes of statistical inference such as Fisherian, Neymanian or Bayesian could be implemented^42^. Here, we take a Neymanian perspective where the potential outcomes are assumed to be fixed and the treatment assignment is the basis of inference. Our goal is to measure the average causal effect for the sample of matched units. We assume that each of the two units of a matched pair *j* has two potential concentrations for an air pollutant. If we were able to observe these potential outcomes, we could simply measure the effect of North-East winds on air pollutant concentrations by computing the finite-sample average treatment effect for matched treated units $\tau _{\text {fs}}$. We would first compute for each pair the mean difference in concentrations and then average the differences over the *J* pairs. While we only observe one potential outcome for each unit, we can nonetheless estimate $\tau _{\text {fs}}$ with the average of observed pair differences ${\hat{\tau }}$:

$$\begin{aligned} {\hat{\tau }} = \frac{1}{J}\sum _{j=1}^J(Y^{\text {obs}}_{\text {t},j}-Y^{\text {obs}}_{\text {c},j}) = {\overline{Y}}^{\text {obs}}_{\text {t}} - {\overline{Y}}^{\text {obs}}_{\text {c}} \end{aligned}$$

Here, the subscripts *t* and *c* respectively indicate if the unit in a given pair is treated or not. Since there are only one treated and one control unit within each pair, the standard estimate for the sampling variance of the average of pair differences is not defined. We can however compute a conservative estimate of the variance^22^:

$$\begin{aligned} \hat{{\mathbb {V}}}({\hat{\tau }}) = \frac{1}{J(J-1)}\sum _{j=1}^J(Y^{\text {obs}}_{\text {t},j}-Y^{\text {obs}}_{\text {c},j} - {\hat{\tau }})^{2} \end{aligned}$$

We finally compute an asymptotic 95% confidence interval using a Gaussian distribution approximation:

$$\begin{aligned} \text {CI}_{0.95}(\tau _{\text {fs}}) =\Big ( {\hat{\tau }} - 1.96\times \sqrt{\hat{{\mathbb {V}}}({\hat{\tau }})},\; {\hat{\tau }} + 1.96\times \sqrt{\hat{{\mathbb {V}}}({\hat{\tau }})}\Big ) \end{aligned}$$

The obtained 95% confidence interval gives the set of effect sizes compatible with our data^43^.

#### Stage 4: Sensitivity analysis

The fourth step of our causal inference pipeline is to explore how sensitive our analysis is to violation of the assumptions it relies upon. We carry out three types of robustness checks.

First, we make the strong assumption that the treatment assignment is as-if random: winds blowing from the North-East occur randomly conditional on a set of measured covariates. Other researchers could however argue that we fail to adjust for unmeasured variables influencing both the occurrence of North-East winds and air pollutant concentrations. Within matched pairs, these unobserved counfounders could make the treated day more likely to have wind blowing from the North-East than the control day. We therefore implement the quantitative bias analysis, also called sensitivity analysis, that was developed by^21,30^. It allows us to explore how our results would be altered by the effect of an unobserved confounder on the treatment odds, denoted by $\Gamma$. In our matched pairwise experiment, we assume that within each pair, control and treated days have the odds to see the wind blowing from the North-East: the odds of treatment is such that $\Gamma =1$. The quantitative bias analysis allows to compute the 95% confidence intervals obtained for different values of bias the unmeasured confounder has on the treatment assignment. For instance, if we assume that an unmeasured confounder has a small effect on the odds of treatment (i.e., for a $\Gamma >1$ and close to 1) but the resulting 95% confidence interval becomes completely uninformative, it would imply that our results are highly sensitive to hidden bias. Conversely, if we assume that an unmeasured confounder has a strong effect on the odds of treatment (i.e., for a large $\Gamma$) and we find that the resulting 95% confidence interval remains similar, it would imply that our results are very robust to hidden bias. In a complementary manner, we also check whether unmeasured biases could be present by using the first daily lags of air pollutant concentrations as control outcomes^44^. If our matched pairs are indeed similar in terms of unobserved covariates, the treatment occurring in *t* should not influence concentration of air pollutants in $t-1$.

Second, for many matched pairs, air pollutant concentrations were imputed using the chained random forest algorithm^39^. We check whether the results are sensitive to the imputation by re-running the analysis for the non-missing concentrations.

Third, we make sure that the treatment assignment within pairs was effective to increase the precision of estimates. We compare the estimate of the sampling variance of a pairwise randomized experiment to the one of a completely randomized experiment. If the estimate of sampling variability for the pairwise experiment is smaller than the estimate of sampling variability for a complete experiment, it means that our matching procedure was successful to match similar units within pairs compared to randomly selected units^22^.

## Results

### Performance of the matching procedure

Our initial dataset consists in 4018 daily observations, divided into 912 treated units and 3106 control units. The matching procedure results in 121 pairs of matched treated-control units—only 13% of treated units could be matched to similar control units given the constraints we set. In the supplementary materials (https://lzabrocki.github.io/design_stage_wind_air_pollution/4_comparing_initial_to_matched_data.html), we show that the matched sample has different characteristics from the initial sample: observations belong more to the period ranging from May to October, their average temperature is higher and their relative humidity is lower.

**Figure 4:**
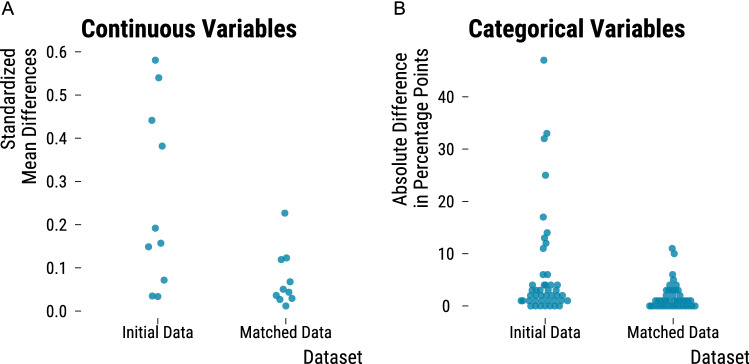
Overall balance improvement in continuous and categorical covariates . In Panel (**A**), we plot, before and after matching, the absolute standardized differences in continuous covariates between treated and control groups. Each blue dot represents an absolute mean difference for a given covariate. In panel (**B**), we plot, before and after matching, the absolute difference in percentage points for categorical covariates.

In Fig. 4, we display how the balance of continuous and categorical covariates improves after the matching procedure. Blue dots represent either the absolute mean differences between treated and control units for continuous variables or the absolute differences in percentage points for categorical variables. For continuous covariates, the average standardized mean differences between treated and control days is 0.26 before matching and reduces to 0.07 after the procedure. For categorical covariates, the average difference in percentage points diminishes from 6.2 to 1.8 after matching. Our matching procedure therefore leads to a consequent reduction of our sample size but allows us to compare treated units that are more similar to control units. A complete analysis of the balance improvement for each covariate is available in the supplementary materials (https://lzabrocki.github.io/design_stage_wind_air_pollution/6_checking_balance_improvement.html).

### North-east wind effects on air pollutant concentrations

**Figure 5:**
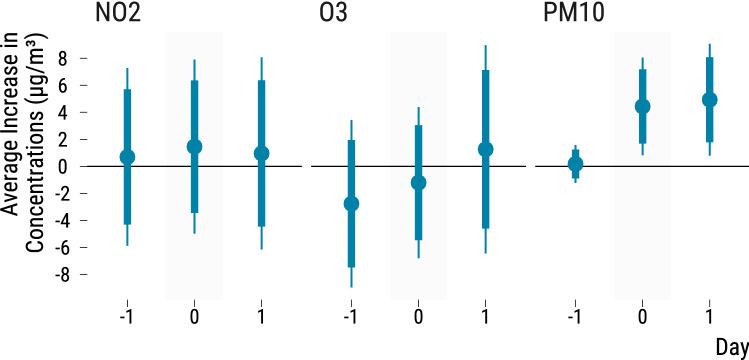
Effects of North-East winds on air pollutant concentrations . In each panel, we plot the estimated effects of North-East winds on air pollutant concentrations for the previous, current and following days. Point estimates are depicted by blue points; blue thick lines are 95% confidence intervals and thin lines are 99% confidence intervals. The 95% and 99% confidence intervals associated with the estimated average difference in PM$_{10}$ in the first lag are smaller than other intervals for the following days since we added a constraint in the matching procedure for this lag of the air pollutant.

For each air pollutant, we plot in Fig. 5 the estimated average difference in concentration ($\upmu \hbox {g}/\hbox {m}^{3}$) between North-East winds and other wind directions. We also display the estimated differences for the previous day and the following day. Thick lines represent the 95% confidence intervals while thin lines are the 99% confidence intervals. The third panel of Fig. 5 confirms the exploratory analysis of the polar plot. When wind blows from the North-East, PM$_{10}$ concentrations increase by 4.4 $\upmu \hbox {g}/\hbox {m}^{3}$, with the lower and upper bounds of the 95% confidence being respectively equal to an increase by 1.7 $\upmu \hbox {g}/\hbox {m}^{3}$ and 7.2 $\upmu \hbox {g}/\hbox {m}^{3}$. The estimated difference represents an 18% increase in the average concentration of PM$_{10}$. We also observe a positive difference of 25% in PM$_{10}$ concentrations the following day (point estimate of 4.9; 95% CI 1.8, 8.1).

North-East winds do not seem to influence NO$_{2}$ (point estimate of 1.5; 95% CI − 3.4, 6.4), and O$_{3}$ (point estimate of − 1.2; 95% CI − 5.5, 3.1) concentrations on the current day. This is also the case for the concentrations of these two air pollutants on the following day.

Regarding the effects of North-East winds on PM$_{2.5}$, we restrain our analysis to pairs without missing concentrations. For the current and following days, we respectively find an average increase of 1.4 $\upmu \hbox {g}/\hbox {m}^{3}$ (95% CI − 0.6, 3.4) and 2.7 $\upmu \hbox {g}/\hbox {m}^{3}$ (95% CI 0.8, 4.5). These point estimates respectively represent a 8.8% and a 17% relative increases in PM$_{2.5}$ concentrations.

### Sensitivity analysis

Our quantitative bias analysis reveals that if we have failed to adjust for an unobserved confounder twice more common among treated days, the resulting 95% confidence intervals for the estimated effects of North-East winds on PM$_{10}$ would be equal to (0.5, 9) for the current day and to (− 0.2, 10) for the the following day. Confidence intervals are still consistent with mostly positive effects but are relatively wide. As a complementary test for unobserved confounders, we also check that the occurrence of North-East winds on the current day does not have any effect on concentrations measured in the previous day. Reassuringly, for NO$_{2}$ and O$_{3}$, 95% confidence intervals do not suggest clear negative or positive average differences in concentrations as shown in Fig. 5 (for PM$_{2.5}$, the estimated average difference is − 0.1 $\upmu \hbox {g}/\hbox {m}^{3}$ (95% CI − 1.2, 1)).

In the supplementary materials (https://lzabrocki.github.io/design_stage_wind_air_pollution/7_analyzing_results.html), we check whether the imputation of missing air pollutant concentrations did not drive our results. For NO$_{2}$, O$_{3}$ and PM$_{10}$, 13%, 8% and 7% of concentrations were respectively imputed. We replicate our analysis on the subset of pairs without missing observations: point estimates remain very similar but confidence intervals are a bit larger due to the sample size loss. This robustness check implies that our imputation did not bias our estimates.

Finally, the pairwise design of our hypothetical experiment does not help increase the precision of the estimated differences in P$M_{10}$ concentrations. The standard error under a completely randomized assignment is equal to 1.35 while the one of a pairwise randomized assignment is 1.4. The pairwise design however increases the precision estimates for O$_{3}$ by 23% for O$_{3}$ but decreases the precision by 42% for NO$_{2}$.

## Discussion

In our study, we follow a causal inference pipeline to craft a hypothetical experiment for measuring the effects of North-East winds on daily particulate matter concentrations in Paris. Our constrained pair matching algorithm enables us to find the subset of treated days that were similar to control days for a set of calendar and weather confounding factors. Compared to a statistical adjustment based on a multivariate regression model, matching is non-parametric and avoids to extrapolate to units without empirical counterfactuals. At the very heart of this method, graphical displays of covariates balance allow to check in a transparent manner whether the as-if random distribution of the treatment was achieved conditional on observed confounders. We were surprised that covariates balance could only be achieved for 13% of treated units. It would be an interesting question for future research to see if alternative methods such as cardinality matching or bayesian additive regression trees lead to similar results^45–47^. The relevant structure of the hypothetical experiment to target should also be of interest since our pair matching algorithm failed to increase the precision of estimates compared to a completely randomized assignment of the treatment.

The difficulty to find similar treated and control units could lead researchers interested in the acute health effects of air pollution to worry that instrumental variable strategies exploiting wind patterns and based on multivariate regression models might suffer from extrapolation bias^10,27^. In the supplementary materials (https://lzabrocki.github.io/design_stage_wind_air_pollution/7_analyzing_results.html), we show that results based on an outcome regression approach, even if they are based on the entire sample, are consistent with those found with the matched data. This may increase the confidence in the capability of a multivariate regression model to correctly extrapolate. Matching estimates are however much less precise. Further research is therefore needed to better understand if improving the design stage of instrument variable studies with matching methods is feasible given the small sample size it entails^48–51^. If it is the case, could matching methods actually lead to different results^52–54^?

In addition to providing evidence on the effective sample size for which covariates balance was achievable, our study was the occasion to assess whether the estimated effects of North-East wind on particulate matters were robust hidden bias. It would require an unmeasured confounder twice more common among treated days to raise doubt on the direction of the estimated effects. This raises our confidence in the assumption that North-East wind are also randomly distributed according to unobserved variables. To the best of our knowledge, this assumption was waiting to be quantitatively evaluated. This could be explained by the fact that the sensitivity analysis we rely on was developed for pairwise matched data^30^. As an alternative, researchers wishing to keep working with a regression approach could implement the new method developed by^55,56^.

Finally, our study presents two main limits regarding the improvement of the design stage of air pollution studies based on wind directions. The first limit concerns the definition of the contrast of interest, that is to say the difference of air pollutant concentrations between North-East winds and other wind directions. If this comparison is easy to understand, the treatment we defined is not manipulable contrary to those found in randomized controlled trials. It might lack a certain appeal to policy-makers as our estimates only indicate whether North-East winds lead to higher particulate matter concentrations than other wind directions^57,58^, without determining the origin of the sources emitting the air pollutant. To overcome this limit, a study exploiting variations in wind directions should be combined with a clear shock on one of the sources emitting an air pollutant. For instance, in a recent paper in Southern California^34^, it was shown that Santa Ana winds have a predominant ventilation effect on PM$_{2.5}$ but when inland wildfires occur, Santa Ana winds are instead increasing PM$_{2.5}$ levels on the coast.

The second limit revolves around the assumption that, for wind direction to be a valid instrument, its effects on a health outcome must be fully mediated by a single air pollutant^7–9^. As recognized by researchers, studies exploiting wind patterns could violate this assumption if changes in wind direction affect simultaneously several air pollutants. In our study, once the data are matched, it seems that North-East winds only influence particulate matter, which could reinforce the credibility of the assumption. Yet, this should not be always the case as it would be highly dependent on the city and air pollutant investigated. Methodological work is much needed to understand in which cases the air pollutants co-variance structure could lead to biased dose-response. In a recent work^59^, propose to run a multi-pollutant model where each air pollutant concentration is predicted by selecting the optimal set of instrumental variables using least absolute shrinkage and selection operator (lasso). The authors show that results of an instrumented multi-pollutant model can be very different from those found by single-pollutant models. It remains to be studied if matching could also help limit this well-known issue.
